# Improving Cognitive Performance of 9–12 Years Old Children: Just Dance? A Randomized Controlled Trial

**DOI:** 10.3389/fpsyg.2019.00174

**Published:** 2019-02-06

**Authors:** Vera van den Berg, Emi Saliasi, Renate H. M. de Groot, Mai J. M. Chinapaw, Amika S. Singh

**Affiliations:** ^1^Department of Public and Occupational Health, Amsterdam Public Health Research Institute, Amsterdam UMC, Vrije Universiteit Amsterdam, Amsterdam, Netherlands; ^2^Welten Institute – Research Centre for Learning, Teaching and Technology, Open University of the Netherlands, Heerlen, Netherlands; ^3^Department of Complex Genetics, School for Nutrition, Toxicology and Metabolism, Faculty of Health, Medicine and Life Sciences, Maastricht University, Maastricht, Netherlands

**Keywords:** physical activity, exercise, selective attention, inhibition, memory, aerobic fitness, MVPA, children

## Abstract

Exercise is assumed to have positive effects on children’s cognitive performance. However, given the inconclusive evidence for the long-term effects of exercise, it is difficult to advice schools on what specific exercise programs can improve children’s cognitive performance. In particular, little is known about the effects of small exercise programs that may be feasible in daily school practice. Therefore, we assessed the effects of a 9-weeks program consisting of daily exercise breaks on children’s cognitive performance, aerobic fitness and physical activity levels. We conducted a cluster-randomized controlled trial in 21 classes of eight Dutch primary schools. A total of 512 children aged 9–12 years participated. The exercise intervention had a duration of 9 weeks and consisted of a daily 10-min classroom-based exercise break of moderate to vigorous intensity. Before and after the intervention, we used four cognitive tasks (i.e., the Attention Network Test, Stroop test, d2 test of attention and Fluency task) to measure children’s cognitive performance in domains of selective attention, inhibition and memory retrieval. In addition, we measured aerobic fitness with a Shuttle Run test and physical activity during school hours by accelerometers. We analyzed data using mixed models, adjusting for baseline scores, class and school. After 9 weeks, there were no intervention effects on children’s cognitive performance or aerobic fitness. Children in the intervention group spent 2.9 min more of their school hours in moderate to vigorous physical activity as compared to the children in the control group. In conclusion, daily 10-min exercise breaks in the classroom did not improve, nor deteriorate cognitive performance in children. The exercise breaks had no effect on children’s fitness, and resulted in 2.9 min more time spent in moderate to vigorous physical activity during school hours. Daily exercise breaks can be implemented in the classroom to promote children’s physical activity during school time, without adverse effect on their cognitive performance.

## Introduction

The assumed positive relationship between exercise and cognitive performance is widely used to advocate in favor of increasing exercise opportunities in schools (e.g., Erwin et al., 2012; Webster et al., 2015; Savina et al., 2016). In particular, since cognitive performance in domains such as selective attention, inhibition, working memory and cognitive flexibility, has been shown to be important for children’s academic performance (Stevens and Bavelier, 2012; Jacob and Parkinson, 2015). However, recent systematic reviews and meta-analyses have shown that the evidence for the long-term effects of structured exercise programs on children’s cognitive performance is inconclusive; some studies report positive effects, while others report no effects (see for reviews Donnelly et al., 2016; Li et al., 2017; Watson et al., 2017; Singh et al., 2018). Nevertheless, it can be concluded that increasing the time spent on exercise in school at the cost of academic lessons does not negatively impact children’s cognitive performance (Donnelly et al., 2016; Singh et al., 2018).

Recently, an international expert panel indicated that there is a need for more well-designed, randomized controlled (RCT) trials to gain better insight in the causal effects of exercise on cognition (Singh et al., 2018). In addition, the experts highlighted the importance of elucidating the characteristics of exercise interventions that may improve cognitive performance. Due to substantial heterogeneity in interventions (e.g., duration, frequency, content), it is difficult to advise schools on the optimal form of exercise interventions to improve children’s cognitive performance (Donnelly et al., 2016; Watson et al., 2017; Singh et al., 2018).

The vast majority of studies that examined the long-term effects of exercise on cognitive performance of children have implemented extensive exercise interventions with durations of 30–60 min per session, mostly delivered three to five times a week (Donnelly et al., 2016; Alvarez-Bueno et al., 2017; Singh et al., 2018). However, it seems unlikely that such time-consuming programs will be implemented on a large scale in real-life daily school practice. Several qualitative studies have reported that time constraints are perceived as a major barrier that limit the opportunities for physical activity and exercise in schools (Howie et al., 2014b; McMullen et al., 2014; Stylianou et al., 2015; Dinkel et al., 2017; van den Berg et al., 2017). In addition, teachers indicate that it would only be feasible to implement short exercise bouts with a maximum duration of 5–10 min (Howie et al., 2014b; van den Berg et al., 2017).

Previous studies have focused on the *acute*, or immediate, effects of relatively short exercise bouts on cognitive performance, such as attention, inhibition and working memory (e.g., Niemann et al., 2013; Howie et al., 2015; van den Berg et al., 2016). Several systematic reviews and meta-analyses concluded that *overall*, single moderate to vigorous exercise bouts with a minimum duration of 10 min can have small to moderate acute positive effects on children’s classroom behavior (i.e., time-on-task) (Watson et al., 2017; Daly-Smith et al., 2018), selective attention (Chang et al., 2012; Janssen et al., 2014; de Greeff et al., 2018), and executive functioning (Chang et al., 2012; Verburgh et al., 2014; Donnelly et al., 2016; Ludyga et al., 2016). However, it is still unclear whether these acute effects accumulate over time, i.e., if implementing short exercise bouts on a regular basis can improve children’s cognitive performance after weeks or months.

Several potential mechanisms underlying the effects of exercise on cognition have been discussed in the literature. For example, acute effects of exercise have been related to increased blood flow (Ogoh and Ainslie, 2009), increased release of neurotrophic factors, such as brain derived neurotropic factor (BDNF) and insulin-like growth factor-1 (Piepmeier and Etnier, 2015), increased arousal levels (McMorris and Hale, 2015), and increased activity in certain brain areas (Budde et al., 2008). Mechanisms of chronic exercise effects include increased availability of growth factors (e.g. BDNF), development of new blood vessels and neurons, changes in brain volume, increased efficiency of neural networks, and increased physical fitness (see for reviews Hillman et al., 2008; Huang et al., 2014; Fernandes et al., 2017). In addition, chronic exercise is suggested to improve self-control, which is important for self-regulation and functioning of higher cognitive functions (Audiffren and André, 2015). Some of the above-mentioned mechanisms may explain cumulative effects of acute exercise. For example, acute exercise-induced elevations of BDNF have been shown to be augmented by repeated exercise, resulting in increased resting levels of BDNF important for cognitive improvements and brain changes (see for a review Huang et al., 2014). Furthermore, several acute and long-term studies have shown that cognitively demanding exercise (e.g., coordinative exercise, team games) can improve cognitive performance to a higher extent than mere repetitive aerobic exercise (e.g., Budde et al., 2008; Koutsandreou et al., 2016; Schmidt et al., 2016), likely due to the inherent motor and cognitive demands (Best, 2010; Tomporowski et al., 2015). Acute cognitively demanding exercise requires high cognitive effort due to exercise complexity and changing circumstances, which may provide long-term improvement of self-control capacities and cognitive functioning (Best, 2010; Audiffren and André, 2015). This type of exercise could also result in higher intervention compliance, since challenge and variety seem important for children’s exercise motivation (e.g., Martins et al., 2015).

To the best of our knowledge, no previous studies investigated the long-term effects of short exercise breaks (i.e., 10 min) in the classroom on cognitive performance of preadolescents (aged 9–12 years). Costigan et al. (2016) examined the effects of two 8-week interventions, in which 8- to 10-min exercise bouts consisting of (1) high intensity aerobic exercises or (2) high intensity combined aerobic and strength exercises were implemented three times per week in 14–16 years old adolescents. The exercise bouts were implemented once a week during recess and twice a week as part of the regular physical education (PE) classes. The authors found no significant differences in executive functioning between the intervention groups and the control group that followed the regular PE classes (Costigan et al., 2016). Little contrast in the amount of additional exercise in the three groups and the absence of measures to compare adolescent’s physical activity levels limit conclusions about the exercise related effects on cognitive performance. Furthermore, the authors indicated that the relatively small sample (*N* = 65) from one secondary school limits the generalizability of their results (Costigan et al., 2016).

To fill this gap, we conducted a cluster RCT trial to investigate the effects of a 9-week exercise break program on cognitive performance of 9–12 years old Dutch primary school children. The intervention consisted of one daily, classroom-based 10-min exercise break in which children were asked to mimic dance movements (i.e., aerobic exercise with coordinative and cognitive demands). The intervention was implemented within the school curriculum, as it has been shown that curricular exercise programs can result in stronger effects on cognition compared to programs that are implemented outside school hours (Alvarez-Bueno et al., 2017). Moreover, Dutch teachers have indicated that classroom-based physical activity is most feasible in daily school practice (van den Berg et al., 2017).

We examined the effects of the intervention on selective attention, inhibition, and semantic memory retrieval, since these cognitive domains are associated with children’s academic performance (Rueda et al., 2010; Stevens and Bavelier, 2012). As secondary outcomes, we measured children’s aerobic fitness and their physical activity levels during school hours. Given the earlier reported *acute* effects of short exercise bouts, we hypothesized that implementing a daily exercise break will have a positive effect on children’s cognitive performance after 9 weeks.

## Materials and Methods

### Sample Size Calculation

We used G^∗^power 3.1.9.2 (Faul et al., 2007) to calculate the required sample size. In line with earlier studies and meta-analytic findings, we expected to find a small to medium effect of our exercise intervention on children’s cognitive performance (e.g., Schmidt et al., 2015; Costigan et al., 2016; Vazou et al., 2016). The sample size calculation revealed that we needed to include a total of 404 participants (*N* = 202 per group) to detect a small to medium effect (*f* = 0.18) of the intervention on children’s cognitive performance, with a power of 95% (two-sided testing at α = 0.05).

### Recruitment of Participants

We approached a convenience sample of regular primary schools from the network of our research group by email and personal contact. Twenty-three schools across the Netherlands received an information letter and were asked to respond if they were interested to participate. We included schools that were willing to participate with a minimum of two classes. For feasibility reasons, we decided to stop the inclusion after eight schools agreed to participate. Two schools declined due to busy school schedules and one school was excluded since they had only one class available. Twelve schools did not respond, but were neither followed-up since we reached the required sample size with schools that responded to our first invitation.

All children in grades 5 and 6 (*N* = 549) were invited to participate. Children and their parents/caregivers received an information letter about the study, including an informed consent form. In consultation with the schools it was decided that all children participated in the intervention/control program as part of the regular school curriculum. Permission of at least one parent/caregiver and children of 12 years and older was required to participate in the measurements. We received informed consent of 512 children (93%), who were included in the study. The study was approved by the Medical Ethical Committee of the VU University Medical Center Amsterdam [2014.363].

### Study Design, Randomization and Blinding

We conducted a cluster RCT. An independent statistician randomly assigned the participating classes to the intervention (*N* = 11) or control group (*N* = 10). Randomization was performed in R using block randomization with blocks of size 2. The randomization was stratified by school and grade for the schools in which multiple classes of the same grade took part. Randomization for the remaining schools was done by randomly assigning the 5th grade to one of the two conditions (with the 6th grade automatically receiving the alternative). This procedure ensured that in each school there were both control and intervention classes and that number of control and intervention classes was balanced between the two grades. The randomization took place after the pretest measurements to ensure that all children, teachers and researchers were blinded. Two members of the research team remained blinded the entire experiment and acted as test administrators at the posttest measurements.

### Procedure

**Figure 1:**
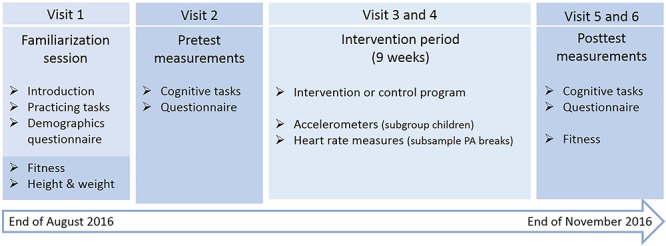
Overview and timeline of the study.

Before the experiment, we trained the research team to conduct the measurements following a standardized protocol. We visited each class six times (see Figure 1). The first visit consisted of a familiarization session in which we introduced the study and explained all measurement procedures. Children received detailed instructions about four cognitive tasks and practiced all tasks to make sure they understood them well. Furthermore, children filled out a demographics questionnaire and we measured their baseline height, weight, and fitness. During the second visit, we conducted the baseline measurements (pretest) in which the cognitive tasks and a questionnaire were administered. We randomly assigned the children in each class to group A and B, stratified by gender (10–15 children per group, depending on class size). Group A started in the classroom where two paper-and-pencil cognitive tasks and the questionnaire were administered, while group B started in a separate room where two computerized cognitive tasks were administered on laptops. Halfway the test session, the groups switched rooms and continued with the other half of the measurements. In one school (*N* = 2 classes) there was no private room available, so we administered the paper-and-pencil tasks with the entire class, and divided the classroom in two testing areas for the laptop tasks and questionnaire. The week after the baseline measures, the classes started with the 9-week intervention/control program. The third and fourth visit were scheduled during the intervention period to: (1) hand out accelerometers in a subgroup of children, and (2) measure the exercise intensity of one exercise break in the intervention classes. After the intervention period, we conducted the post-intervention measurements (fifth visit: posttest), which were identical to the pretest and scheduled at the same day of the week and time of the day. To avoid contamination of the effects by possible acute exercise effects, we instructed all teachers not to perform an exercise break on the measurements days. During the sixth visit we measured children’s fitness again. After the experiment, all children received a small symbolic present for their participation.

### Intervention Program

The intervention lasted 9 weeks and consisted of one moderate to vigorous intensity exercise break per school day. The intervention duration was chosen for feasibility reasons. A period of 9 weeks best fitted the school’s year schedules and was relatively short, by which we aimed to keep compliance high during the entire intervention period.

Each exercise break lasted approximately 10 min and consisted of three ‘Just Dance’ videos (Ubisoft, free available via YouTube). In the videos, a figure performs a dance which the children are asked to mimic. Our choice for Just Dance videos and the exercise duration of 10 min was based on the acute exercise literature combined with the preferences of Dutch teachers and children in the upper grades of primary school. The exercise literature indicates that moderate-to-vigorous exercise bouts need to have a duration of at least 10 min to exert acute cognitive improvements (Howie et al., 2014a, 2015; Daly-Smith et al., 2018). Teachers have indicated that they prefer additional PA in school to be classroom-based, easy to implement (i.e., requiring little preparation time) and up to a maximum of 10 min (van den Berg et al., 2017). Accordingly, many teachers in the Netherlands already use Just Dance in the classroom setting. A recent study of our group revealed that one of the ideas of children to increase PA in school, that matched the preferences of teachers, is to implement short exercise breaks during classroom time, for example Just Dance (van den Berg et al., 2018). Teachers received an instruction sheet with a password to access the exercise breaks via a secured webpage. The exercise breaks were performed in the classroom and we asked teachers to make sure that all children kept moving. The exercise break program was performed in addition to the regular PE classes.

The exercise breaks were selected based on a pilot study, in which we tested 83 Just Dance videos once (100%) or twice (67%) in 31 grades 5 and 6 of 14 primary schools (unpublished data). Children (*N* = 766) wore heart rate monitors to determine the mean exercise intensity of each video and we asked them to rate the videos on being fun and difficult, respectively. In addition, we observed the feasibility of performing the dances in the classroom. The pilot resulted in the selection of 55 suitable videos that were used to compose 45 different exercise breaks used in the current study. To ensure variety in the program, each exercise break consisted of a unique combination of three videos. Each video returned two or three times during the 9 weeks period, each time combined with two other videos.

### Control Program

The control program consisted of nine educational lessons, lasting 10–15 min, one for each week of the experiment. The lessons were unrelated to the core school curriculum. We composed the lessons using information and educational videos on topics related to the body, exercise and/or sports for 9–12 year olds (free available online; see for example NTR, 2012). During six lessons children watched a short 5-min video (e.g., about agility in gymnasts, the role of balance in sports, or endurance in runners) and were asked to answer four to five questions based on what they learned from watching the video. In three lessons, children were asked to read one page of written information, for example about bones, joints and muscles in the body or about the respiratory system, and to answer five questions based on this information. The teacher discussed the answers with the entire class at the end of each lesson. Teachers received an instruction card including a password to access a secured webpage with the instructions, videos, worksheets and answers for each lesson. During the experiment, children in the control group followed the regular PE classes.

### Measures and Measurement Instruments

#### Demographics and Anthropometrics

Children self-reported their birth date, gender and sports participation. The questions on sports participation were derived from the ENERGY-child questionnaire, showing good to excellent test-retest reliability (ICC’s: 0.68–1.00) and moderate to excellent construct validity (ICC’s: 0.51–1.00) (Singh et al., 2011). The Dutch version of the Harter’s Self Perception Profile for Children was administered to measure children’s perceived competence in five domains (scholastic, social, athletic, physical appearance, behavioral conduct) and their perceived global self-worth (Veerman et al., 1997). This questionnaire has been shown to have sufficient construct validity and good test–retest reliability (ICC’s ≥ 0.84) in 8–14 years old Dutch children (Muris et al., 2003; Egberink and Vermeulen, 2018a). Parents self-reported their highest completed educational level, which was used as a proxy measure of socio-economic status.

We asked teachers to provide standardized test scores of the children on reading comprehension, orthography and arithmetic. Scores were obtained from the standardized and norm-referenced CITO test battery (Hollenberg and Van Der Lubbe, 2017), which most schools in the Netherlands administer twice a year to assess and track children’s academic performance. After the experiment, teachers provided information on children with special educational needs (e.g., ADHD, autism spectrum disorders, learning disorders).

We measured children’s body height (cm) and weight (kg) in sport clothes without shoes, using a Leicester Height Measure Mk II (Harlow Healthcare, United Kingdom) and a Seca weighting scale (Seca Instruments, Frankfurt, Germany). The Body Mass Index (BMI) of each child was calculated with the formula: [weight (kg)/height (m)^2^].

#### Intervention Integrity

Each class received a calendar-poster that was attached to the classroom wall and remained visible during the intervention. We asked teachers and children to put a sticker on the poster each time they performed an exercise break (intervention group) or an educational lesson (control group). The poster served as a reminder to implement the program, as well as a measure of intervention integrity. We calculated the percentage of exercise breaks that were conducted, with 45 exercise breaks equaling 100% implementation. Halfway the intervention, we asked teachers to report potential implementation problems. In case of problems, we gave advice and encouraged teachers to implement as many exercise breaks or educational lessons as possible.

#### Exercise Break Intensity

We assessed the intensity of a subsample of exercise breaks by monitoring heart rate (11 exercise breaks; one per intervention class). All children were fitted with a Polar H7 Bluetooth heart rate monitor that was connected to the Polar Team App (Polar Electro Oy, Finland) in which the mean heart rate of each child was stored. Exercise intensity was calculated as percentage of the maximum heart rate: (mean heart rate/maximum heart rate)^∗^100. The maximum heart rate was measured during the Shuttle Run test (see Aerobic Fitness).

### Primary Outcomes: Cognitive Performance

We measured cognitive performance with two paper-and-pencil tasks, i.e., the d2 Test of Attention and the Fluency Task, and two computerized tasks, i.e., the Stroop Color-Word task and Attention Network Task (ANT) using E-prime 1.2 Software (Psychology Software Tools, Pittsburgh, PA, United States). During the pre- and posttest, children received standardized verbal and written instructions and made a few practice trials (d2 test, Stroop, ANT). Two trained and blinded test instructors gave task instructions for all tests and kept track of time in case of the d2 test and Fluency task. During the tests, two to three members of the research team each supervised a small group of children and made notes. We instructed the children to work quietly, individually, and as fast and accurately as possible. The order in which the tests were administered was counterbalanced and randomized, stratified by gender, grade and intervention/control group. The order of tests was identical during the pre- and posttest and each child made all tests on the same laptop.

#### d2 Test of Attention

The d2 test was used to measure selective attention (Brickenkamp and Oosterveld, 2012). The construct validity of the d2 test has been rated as sufficient (Egberink and Vermeulen, 2018c). The reliability has been rated as good, with moderate to high test-retest reliability in 10–13 years old Dutch children (*r* = 0.79–0.83) (Brickenkamp and Oosterveld, 2012; Egberink and Vermeulen, 2018c).

The d2 test consists of one page with fourteen lines, each consisting of 47 characters ‘d’ and ‘p’ with one to four dashes displayed above and/or below. We instructed the children to mark as much letters ‘d’ with a total of two dashes (‘d2’) as possible, while ignoring the other characters. They had to work from the left to the right, with a time limit of 20 s per line. The test instructor gave a signal when to continue with the next line. The total test lasted 4 min and 40 s.

We used the concentration performance (i.e., number of correctly marked d2’s minus the number of incorrectly marked characters) as dependent variable, since this is an objective measure of selective attention (Brickenkamp and Oosterveld, 2012).

#### Fluency Task

We used a paper-and-pencil version of the Verbal Fluency task (Mulder et al., 2006) to measure semantic memory retrieval performance. The validity and reliability of the Verbal Fluency task has been shown sufficient in children and adolescents (Korkman et al., 1998; Egberink and Vermeulen, 2018b).

Children were instructed to write down as many words as possible in the category ‘animals’ within 60 s. The total number of correct words was used as dependent variable.

#### Attention Network Task

We used the short version of the ANT to assess the efficiency of three attentional networks: alerting (i.e., achieving and maintaining an alert state), orienting (i.e., selection of information from sensory input) and executive control (i.e., resolving conflict among responses) (Fan et al., 2002, 2007). Several studies have recommended the use of the ANT in children, as it has been shown a valid instrument to measure their attentional performance (Rueda et al., 2004; Forns et al., 2014). The task was downloaded from the website of the Sackler Institute for Developmental Psychobiology (Sackler Institute for Developmental Psychobiology [SIDP], 2016).

Sets of five horizontal black arrows pointing to the right or left were presented on a white 15-inch laptop screen. Children were instructed to identify the direction of the middle arrow (the ‘target’), by pressing the right mouse button for the right direction and the left mouse button for the left direction. The central target was ‘flanked’ by two lateral arrows on the left and on the right, pointing either in the same direction (congruent; >>>>> or <<<<<) or in the opposite direction (incongruent; >><>> or <<><<). A fixation cross remained visible in the middle of the screen during the task. In two-third of the trials, a warning cue (^∗^) was presented for 200 ms either above or below (spatial cue) or at the place of the fixation cross (center cue) before the stimuli appeared. The total task lasted approximately 12 min and contained three blocks of 48 trials, with 1-min breaks in between.

We calculated the mean reaction time (correct responses only) and accuracy (proportion of correct responses) by the formulas of Fan et al. (2007): Alerting effect = (SCORE no cue – SCORE center cue); Orienting effect = (SCORE center cue - SCORE spatial cue); Conflict effect (executive control) = (SCORE incongruent - SCORE congruent). Larger reaction time scores indicate better alerting and orienting performance, while a smaller value indicates better conflict performance. For accuracy, a larger value indicates better alerting performance, a larger negative value better orienting performance, and a smaller negative value better conflict performance. Reaction times faster than 200 ms were considered as incorrect and excluded from the data analysis (Fan et al., 2007).

#### Stroop Color-Word Task

We used a computerized Stroop Color-Word task to assess children’s inhibitory performance. Computerized versions of the Stroop have been shown to have moderate to good test-retest reliability in children (*r* = 0.50–0.80) (Penner et al., 2012).

During the task, a color-word (the Dutch word for BLUE, GREEN, or RED) was presented on a 15-inch white laptop screen. In the congruent conditions, the color-word was displayed in a similar text color as the meaning of the word (e.g., the word BLUE displayed in a blue text color). In the incongruent conditions, the text color differed from the meaning of the color-word (e.g., GREEN written in a red text color). Children were instructed to press the button ‘1,’ ‘2’ or ‘3’ at the left side of the key board that corresponded to the text color of the color-word. A fixation cross was presented for 1000 ms, followed by the color-word that was presented for 2500 ms. After a child responded, the color-word disappeared. The inter stimuli interval was 4000 ms. The answer options, 1 = GREEN, 2 = BLUE, 3 = RED, remained visible at the bottom of the screen. The task consisted of 105 trials and lasted approximately 9 min.

We calculated the interference score as dependent variable by subtracting the scores of the incongruent from the congruent conditions for both reaction time (correct responses only) and accuracy rates. A smaller interference score indicates better inhibition.

### Secondary Outcomes

#### Aerobic Fitness

We conducted a Shuttle Run test to assess children’s aerobic fitness (Léger et al., 1988). Due to the limited dimensions of the sports halls, all children performed the test over a distance of 18 m instead of 20 m. The highest completed stage was recorded with an accuracy of a half stage and was used to estimate children’s VO_2_max (Léger et al., 1988). All children were familiar with the test and were encouraged by the research team to exert maximum performance. Children wore heart rate monitors (Polar H7, Polar Team App) to determine their maximum heart rate.

#### Physical Activity Levels

We measured children’s PA during the intervention period with GT3x ActiGraph accelerometers (De Vries et al., 2009). In each class, we randomly selected a subgroup of 11–19 children that were asked to wear the device during waking hours for seven consecutive days, including the weekend (mean of 15 children per class; total *n* = 330). We gave children verbal instructions on how to wear the device and provided them and their parents/caregivers with an information sheet including a web-link to an online instruction video. ActiLife 6.13.3 software (ActiGraph, LCC.) was used to initialize the accelerometers and for processing the data (epoch = 15 s).

We calculated children’s PA levels during school hours only. We included children in the data analysis when they wore the accelerometer at least 4 week days (Yildirim et al., 2011). We created a time filter for each school to extract only the exact school hours for analysis (e.g., 08:30 a.m. to 15:00 p.m.). Recess time was included in the analyses, because this is part of a regular school day for both intervention and control group. Non-wear time was defined as having 20 min consecutive zero’s (Yildirim et al., 2011). We used the cut points of Evenson (Evenson et al., 2008) to estimate the time spent in sedentary (0–100 cpm), light (101–2295 cpm), moderate (2296–4011 cpm) and vigorous intensity activity (>4012 cpm), which have been shown to most accurately classify PA intensity levels in children and adolescents (Trost et al., 2011).

### Data Analysis

We performed all statistical analyses in SPSS version 22.0 (IBM SPSS Statistics). Independent *t*-tests and Chi-square tests were used to compare baseline values of the control and intervention group. To test the effect of the intervention, we conducted a separate mixed-model analysis for each cognitive outcome and for aerobic fitness (VO_2_max). The mixed-model included the cognitive outcome or VO_2_max as dependent variable and group (i.e., control or intervention) as fixed factor. Class and school were included as random intercepts. Covariates were the pretest score on the dependent variable, age and/or arithmetic performance. The latter two were included because of group differences at baseline and their expected relationship with the dependent variables. Differences in PA levels between the intervention and control group were also analyzed by mixed-models, with group as fixed factor, class and school as random intercepts, and total wear time as covariate. The level of significance was set at α < 0.05.

We used an intention-to-treat approach, including all children that participated in the study in the data analyses. However, children with a missing pre- or posttest score of the dependent variables or with a missing score on a covariate, were excluded from the respective analysis. In addition, children who did not fully understand or follow the test instructions [i.e., having accuracy rates below chance level (<50%) in the ANT or Stroop task, or indicated by a note of the researchers] were excluded from the respective analysis.

## Results

### Study Population and Descriptive Characteristics

**Figure 2:**
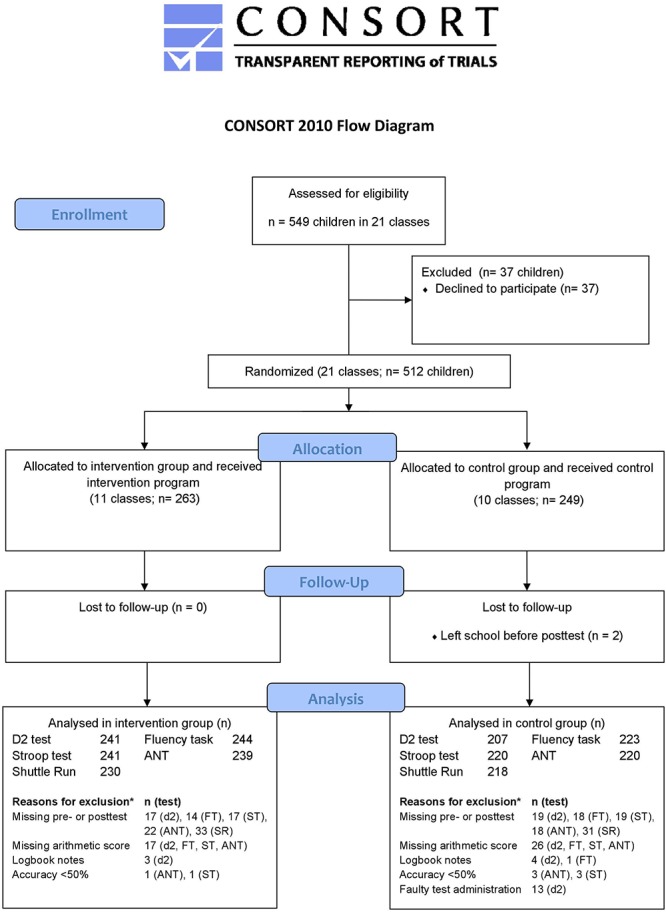
CONSORT flow diagram: progress of participants through the trial. ^∗^Children can be excluded for one or more reasons; e.g., a child with a missing arithmetic score can also have a missing pretest score. d2, d2 test; FT, Fluency task; ST, Stroop test; ANT, Attention Network Test; SR, Shuttle Run test

A total of 510 children between 9 and 12 years old completed the trial (*n* = 2 lost to follow-up). The number of children included in the data analyses ranged from 448 to 467, depending on the outcome variable (Figure 2). A flow diagram including the numbers and reasons for exclusion can be found in Figure 2. In addition to common reasons for exclusion (e.g., absence during the pre- or posttest, missing arithmetic score), we excluded 13 children from the d2 test analysis due to a technical mistake in the test administration by one of the test instructors.

Baseline characteristics of the control and intervention group were similar, except for age and arithmetic performance (see Table 1). There were no significant differences between the groups in pretest scores on any of the outcome variables.

**Table 1:** Descriptive characteristics (means and standard deviations) and group differences.

	Control group (*n* = 249)	Intervention group (*n* = 263)	*p*-value
Age (years)	10.9 (0.7)	10.8 (0.6)	0.01^∗^
Special educational needs (%)	13	19	0.06
Sex (%, boys/girls)	53/47	54/46	0.79
Parental educational level (%)	(*n* = 228)	(*n* = 244)	0.25
-low	0	0.8	
-low to medium	3.6	1.9	
-medium	24.9	28.9	
-high	63.1	61.2	
Height (cm)	(*n* = 244)	(*n* = 259)	
	148.7 (7.2)	147.5 (7.6)	0.06
Weight (kg)	(*n* = 238)	(*n* = 258)	
	38.4 (6.9)	37.7 (7.3)	0.26
BMI	(*n* = 238)	(*n* = 258)	
	17.3 (2.3)	17.2 (2.3)	0.78
Academic performance	(*n* = 242/244/238)	(*n* = 255/258/258)	
-reading comprehension	42.6 (15.1)	40.7 (16.2)	0.17
-orthography	140.2 (7.4)	140.1 (7.0)	0.88
-arithmetic	102.1 (14.7)	98.0 (13.9)	0.00^∗^
Sports participation (hours per week)	(*n* = 244)	(*n* = 258)	
	3.4 (2.0)	3.3 (1.9)	0.78
Self-competence	(*n* = 236/237)	(*n* = 255/257)	
-scholastic	17.3 (3.8)	16.9 (3.5)	0.22
-social	19.1 (3.5)	18.8 (3.4)	0.36
-athletic	19.1 (3.5)	18.9 (3.4)	0.53
-physical appearance	20.0 (3.7)	19.9 (3.8)	0.98
-behavioral conduct	18.3 (3.0)	18.3 (2.9)	0.79
-self-worth	20.7 (3.1)	20.5 (3.6)	0.46
VO_2_max, pretest (ml/kg/min)	(*n* = 236)	(*n* = 253)	
	48.1 (5.0)	48.0 (5.0)	0.84
d2 test, pretest	(*n* = 237)	(*n* = 258)	
	133.9 (22.9)	132.1 (22.2)	0.38
Fluency, pretest	(*n* = 240)	(*n* = 258)	
	10.7 (3.3)	10.8 (3.3)	0.80
Stroop interference, pretest	(*n* = 236)	(*n* = 254)	
-reaction time (ms)	46.2 (69.2)	41.1 (80.2)	0.45
-accuracy (%)	-1.0 (5.4)	-1.7 (5.2)	0.14
ANT pretest, reaction time (ms)	(*n* = 236)	(*n* = 253)	
-Alerting	25.6 (37.3)	24.6 (35.5)	0.78
-Orienting	58.0 (36.5)	61.1 (39.6)	0.37
-Conflict	108.4 (45.4)	105.3 (41.5)	0.43
ANT pretest, accuracy (%)	(*n* = 236)	(*n* = 253)	
-Alerting	0.7 (5.1)	0.8 (4.9)	0.80
-Orienting	-1.5 (4.3)	-1.3 (4.7)	0.70
-Conflict	-5.3 (5.0)	-5.9 (5.8)	0.27

^∗^*Significant group-difference, *p* < 0.05*.

### Intervention Integrity and Exercise Break Intensity

The median of implemented exercise breaks was 89%, which corresponds to 4.4 exercise breaks per week during the 9-week intervention (range: 49–98% across classes). The mean exercise intensity of the subsample of tested exercise breaks was 60% (SD 8.5) of the maximum heart rate.

### Intervention Effects: Cognitive Performance and Fitness

We found no significant differences between the intervention and control group in any of the cognitive outcomes, after controlling for pretest score, age, arithmetic performance, class and school. Children in both groups showed similar patterns of change from pre- to posttest. Thus, the exercise intervention did not improve cognitive performance of the children as compared to the control group. We found no intervention effect on aerobic fitness either. An overview of the mean scores, regression coefficients, 95% confidence intervals, and *p*-values can be found in Table 2.

**Table 2:** Test performance (means, standard errors, and [95% confidence intervals]) and statistics of the mixed model analyses for cognitive performance and fitness.

Dependent variable (posttest)	Control group	Intervention group	Regression coefficient (*SE*)	95% confidence interval	*p*
d2 test	151.2 (0.86)	152.5 (0.80)	1.3 (1.5)	-1.9 – 4.4	0.42
(*n* = 448)	[149.5; 152.9]	[151.0; 154.1]			
Fluency	11.9 (0.17)	11.7 (0.16)	-0.2 (0.2)	-0.7 – 0.2	0.33
(*n* = 467)	[11.6; 12.2]	[11.4; 12.0]			
Stroop interference, reaction time (ms)	41.1 (4.4)	33.4 (4.2)	-7.7 (6.0)	-19.6 – 4.1	0.20
(*n* = 461)	[32.6; 49.7]	[25.2; 41.6]			
Stroop interference, accuracy (%)	-1.8 (0.4)	-1.3 (0.3)	0.5 (0.5)	-0.5 – 1.5	0.29
(*n* = 461)	[-2.5; -1.1]	[-2.0; -0.6]			
ANT alerting, reaction time (ms)	23.5 (2.0)	23.5 (1.9)	0.0 (2.8)	-5.4 – 5.4	0.99
(*n* = 459)	[19.6; 27.4]	[19.7; 27.2]			
ANT orienting, reaction time (ms)	61.6 (2.1)	61.5 (2.0)	1.9 (3.3)	-4.5 – 8.3	0.57
(*n* = 459)	[57.5; 65.6]	[57.6; 65.4]			
ANT conflict, reaction time (ms)	95.6 (2.3)	92.3 (2.2)	-3.9 (3.7)	-11.1 – 3.4	0.29
(*n* = 459)	[91.1; 100.1]	[88.0; 96.7]			
ANT alerting, accuracy (%)	1.0 (0.3)	1.2 (0.3)	0.1 (0.5)	-0.8 – 1.0	0.85
(*n* = 459)	[0.4; 1.7]	[0.5; 1.8]			
ANT orienting, accuracy (%)	-1.5 (0.3)	-1.4 (0.3)	0.1 (0.5)	-0.8 – 1.1	0.77
(*n* = 459)	[-2.2; -0.9]	[-2.0; -0.7]			
ANT conflict, accuracy (%)	-6.4 (0.4)	-5.9 (0.3)	0.6 (0.5)	-0.4 – 1.6	0.24
(*n* = 459)	[-7.1; -5.7]	[-6.6; -5.2]			
VO_2_max (ml/kg/min)	48.8 (0.2)	48.9 (0.2)	0.1 (0.3)	-0.6 – 0.7	0.77
(*n* = 448)	[48.4; 49.2]	[48.5; 49.3]			

### PA Levels

A total of 312 children (95%) had valid wear time and were included in the data analysis. Children in the intervention group spent on average significantly more minutes of their school hours in moderate PA (1.7 min) and moderate to vigorous PA (MVPA; 2.9 min) per day as compared the control group, adjusted for total wear time, class and school (see Table 3).

**Table 3:** Physical activity levels during school hours (means, standard deviations, [95% confidence intervals]) and statistics of the mixed-model analyses.

	Control (*n* = 144)	Intervention (*n* = 168)	Regression coefficient	95% confidence interval	*p*
Sedentary (minutes/day)	228.9 (2.0)	224.6 (1.8)	-2.6 (4.3)	-11.9 – 6.7	0.56
	[225.1; 232.8]	[221.0; 228.2]			
Light PA (minutes/day)	98.2 (1.6)	99.4 (1.5)	-0.3 (3.5)	-7.9 – 7.4	0.95
	[95.0; 101.4]	[96.5; 102.4]			
Moderate PA (minutes/day)	12.6 (0.4)	14.4 (0.3)	1.7 (0.7)	-0.3 – 3.2	0.02^∗^
	[11.8; 13.3]	[13.7; 15.1]			
Vigorous PA (minutes/day)	8.1 (0.4)	9.3 (0.3)	1.2 (0.7)	-0.2 – 2.6	0.09
	[7.3; 8.8]	[8.7; 10.0]			
MVPA (minutes/day)	20.6 (0.7)	23.8 (0.6)	2.9 (1.3)	0.2 – 5.6	0.04^∗^
	[19.3; 22.0]	[22.5; 25.0]			

^∗^*Significant, *p* < 0.05; PA, physical activity; MVPA, moderate to vigorous physical activity. PA levels include the exercise break time in the intervention group*.

## Discussion

Daily exercise breaks did not improve nor harm children’s selective attention, inhibition and semantic memory retrieval performance as compared to the control group. Likewise, there were no effects on children’s aerobic fitness. Children that followed the intervention spent about 3 min more of their school hours in moderate to vigorous PA per day than the children in the control group.

Our results are in line with the study of Costigan et al. (2016) who assessed the effect of two 8-week exercise interventions, consisting of short exercise bouts that were implemented three times a week, on executive functioning in adolescents. Although there were several differences between our study and the study of Costigan and colleagues, such as the sample size (512 versus 65), setting in which the exercise bouts were implemented (classroom versus during recess and PE), exercise intensity (moderate versus high) and age of the participants (9–12 years versus 14–16 years), there were also similarities. In both studies, the exercise intervention lasted approximately 2 months and consisted of bouts of approximately 10 min. Our findings do not confirm our hypothesis that acute effects of short exercise bouts on cognition accumulate over time. It is possible that exercise sessions of longer duration are needed to have beneficial effects on cognitive outcomes. In this respect, Ludyga and colleagues, who evaluated an 8-week school-based exercise program in which children performed a daily 20-min exercise bout, reported improvements in working memory (Ludyga et al., 2018b) and inhibition (Ludyga et al., 2018a). Furthermore, a longer intervention period than 9 weeks might be needed to find effects of 10-min exercise bouts.

We also found no effects on children’s aerobic fitness, which may be explained by our minimal exercise intervention. This finding is in line with several systematic reviews reporting that school-based exercise interventions with long durations and high frequencies are needed to improve children’s aerobic fitness (e.g., Kriemler et al., 2011; Dobbins et al., 2013; Braaksma et al., 2018). Another reason for the lack of cognitive effects might be due to the coordinative requirements of our exercise breaks. Our exercise breaks may have been (too) difficult for the children, thus limiting the time they were active at moderate-to-vigorous intensity, which has been suggested to be important to exert cognitive effects (Chang et al., 2012; McMorris and Hale, 2012). Furthermore, high difficulty levels might have led to substantial cognitive demands/effort during the exercise breaks, depleting children’s cognitive resources and hindering improvements in cognition after exercise. This claim finds support in some earlier acute studies that reported no effects of classroom-based cognitive demanding exercise bouts on selective attention (van den Berg et al., 2016), updating and inhibition (Jäger et al., 2015; Egger et al., 2018), and even negative effects on shifting (Egger et al., 2018) as compared to low cognitive demanding aerobic exercise in children and young adolescents. In contrast, recent meta-analyses reported positive chronic effects of cognitively demanding exercise programs (e.g., Alvarez-Bueno et al., 2017; de Greeff et al., 2018). Therefore, we recommend future research to examine how exactly the effects of acute and chronic cognitively demanding exercise relate to each other. Furthermore, research is needed to gain more insight in the optimal dose of the cognitive demands, taking into account children’s motor- and cognitive development, and exercise characteristics such as difficulty, duration and intensity (Pesce et al., 2013; Egger et al., 2018).

Although the exercise breaks in our study did not result in improvements in cognitive domains of attention, inhibition and memory retrieval, it might be that short exercise bouts contribute to improved academic performance (e.g., maths or language scores) in the long-term via increasing children’s learning efficiency and academic engagement (e.g., improved classroom behavior, motivation) in the lessons following the exercise bouts (Owen et al., 2016). Long-term effects of exercise interventions with a relatively short bout duration on academic performance in children are inconsistent. A recent study of Fedewa et al. (2018) reported small improvements in reading performance of 8–11 year olds who participated in two 5-min aerobic exercise breaks per day for a period of 9 months as compared to children who performed two 5-min exercise bouts in which academic content was integrated (Fedewa et al., 2018). On the other hand, Ahamed et al. (2007) found that implementing a daily 15-min classroom-based exercise break for 16 months did not improve academic performance in children aged 9–11 years. Given the inconsistent findings, more insights need to be gained on the relevance of implementing short exercise breaks for academic purposes. Therefore, we recommend researchers to (1) combine acute as well as long-term measures; (2) include cognitive- as well as academic outcomes; and (3) include an inactive control group.

Our results further revealed that children who participated in the exercise breaks spent 2.9 min more of their school hours in MVPA per day compared to children in the control group. Our findings are in line with an earlier study that found that implementing three 5-min classroom-based exercise breaks per day, increased schoolchildren’s MVPA levels (Drummy et al., 2016). Hence, these results suggest that implementing short exercise breaks in the classroom are one promising way to promote PA in children. The additional time spent in MVPA during school hours in our study, however, does not equal the length of the exercise breaks, i.e., 10 min MVPA per day. This might be due to an underestimation of MVPA during dance movements using accelerometers (van Ekris, personal communication). On the other hand, it could be that children were not (moderate to vigorously) active the entire exercise break. In this respect, our heart rate data showed that the mean intensity of the exercise breaks was at the lower boundary of MVPA (i.e., 60% HR max), indicating that it may be difficult to reach or sustain moderate to vigorous intensity levels in a classroom setting. The low exercise intensity could also be a reason for not finding improvements in cognitive performance (McMorris and Hale, 2012).

Our results have several implications for practice and future research. First, it is important to be aware of the apparent gap between research and practice. Although we found no effects of daily exercise breaks on children’s cognitive performance, teachers have indicated that they experience improved classroom behavior and performance when using short exercise breaks in the classroom throughout the school year (e.g., Carlson et al., 2015). Therefore, it might be important to consider using more ecological valid measurement instruments, such as systematic observations, teacher logs and/or tasks that mimic curricular activities as a more appropriate representation of classroom-related performance (Khan and Hillman, 2014). In addition, measures of academic engagement and enjoyment of academic lessons can provide important additional information as these factors may have a role in the relationship of exercise and cognitive/academic performance (Owen et al., 2016). Second, the number of exercise breaks implemented in our study was relatively high (median of 4.4 per week), suggesting that 10-min exercise breaks in the classroom are feasible to implement in school practice. However, the controlled setting and reminders during the experiment (i.e., poster-calendar, email contact and visits by the researchers) have likely influenced these outcomes. Future studies should therefore evaluate the feasibility of the long-term implementation of short exercise breaks in real-life school practice. Third, it is important to notice that children’s cognitive performance did not deteriorate either. We can therefore conclude that implementing exercise breaks on a daily basis, instead of devoting this time to academic tasks, has no adverse effect on children’s cognitive performance. Lastly, an increase of 3 min MVPA induced by the exercise breaks is small. However, implementing short exercise breaks can be a relatively feasible and easy manner to start increasing PA opportunities in school. In order to increase minutes of MVPA during a school day, we recommend to complement exercise breaks in the classroom with other short and feasible exercise interventions in school, e.g., during recess or integrated in academic lessons. Furthermore, PA is suggested to have beneficial effects for mental health (e.g., depression), well-being, mood, self-esteem, motivation, and social connectedness (Lubans et al., 2016; Biddle et al., 2018). However, the effects of short exercise bouts on before mentioned outcomes is still unknown (Poitras et al., 2016). Therefore, we recommend including these outcome measures in future research on the effects of short exercise bouts. As such, we can gain deeper insight in the benefits of short exercise bouts on several domains important to children’s (academic) development, and thereby strengthening the relevance of short exercise bouts in school.

Our study has several strengths, such as the RCT design, substantial sample size, blinded test administrators, use of objective measurement instruments, high compliance and a high implementation rate. Though, our study had also some limitations. Our population consisted of children of parents with a relatively high educational level, which limits the generalizability of the results. In addition, we have no baseline accelerometer-based measure of PA. However, the intervention and control classes were equally distributed within each school, i.e., representing a similar population, and did not differ on important descriptive characteristics, such as sports participation, aerobic fitness and parental educational level. Another limitation is that we did not assess children’s PA behavior outside school hours. It could be that exercise breaks influenced children’s PA behavior outside school, for example if children liked the Just Dance videos they could have decided to perform them during leisure time as well. Lastly, for practical reasons we used a paper-and-pencil version of the verbal fluency task which adds a motor component to the task. Therefore, the test outcomes also depend on writing speed and the length of the chosen words. In addition, we used a computerized Stroop task which measures interference effects to a somewhat lower extent than interference effects measured by the original oral version of the Stroop task (Penner et al., 2012).

## Conclusion

In sum, we found that implementing a daily 10-min exercise break for a period of 9 weeks in the classroom had no effects on cognitive performance and aerobic fitness of 9–12-year old children. The exercise breaks brought about 3 min more MVPA during school hours. Therefore we conclude that schools can implement the seemingly feasible daily exercise breaks in the classroom to promote PA in children without adverse effects on their cognitive performance.

## Ethics Statement

This study was carried out in accordance with the recommendations of the Medical Ethical Committee of the VU University Medical Center, with written informed consent from all subjects. All subjects gave written informed consent in accordance with the Declaration of Helsinki. The protocol was approved by the Medical Ethical Committee of the VU University Medical Center.

## Author Contributions

VvdB, ES, RdG, MC, and AS conceived and designed the study. VvdB and ES acquired the data. VvdB analyzed the data. VvdB, MC, and AS interpreted the data analyses. VvdB drafted the manuscript. ES, RdG, MC, and AS contributed to critical revision of the draft. All authors read and approved the final manuscript.

## Conflict of Interest Statement

The authors declare that the research was conducted in the absence of any commercial or financial relationships that could be construed as a potential conflict of interest.

## Abbreviations

MVPA: moderate to vigorous physical activity
PA: physical activity.
